# Implications of Celiac Disease in Prospective Corneal Refractive Surgery Patients: A Narrative Review

**DOI:** 10.7759/cureus.65761

**Published:** 2024-07-30

**Authors:** Majid Moshirfar, David G Melanson, Shreya Pandya, Kayvon A Moin, Chad L Talbot, Phillip C Hoopes

**Affiliations:** 1 Ophthalmology, Hoopes Vision Research Center, Draper, USA; 2 Ophthalmology, John A. Moran Eye Center, University of Utah School of Medicine, Salt Lake City, USA; 3 Eye Banking and Corneal Transplantation, Utah Lions Eye Bank, Murray, USA; 4 Ophthalmology, Rocky Vista University College of Osteopathic Medicine, Ivins, USA; 5 Ophthalmology, University of Louisville School of Medicine, Louisville, USA; 6 School of Medicine, American University of the Caribbean, Cupecoy, SXM

**Keywords:** type 1 diabetes mellitus, vitamin a deficiency, retinopathy, ophthalmic manifestations, dry eye, uveitis, guidelines, smile, prk, lasik

## Abstract

Celiac disease (CeD) is a prevalent autoimmune disorder incited by gluten consumption, resulting in intestinal damage. Affecting approximately one in 133 people globally, CeD often remains undiagnosed due to its varied clinical presentations. The prevalence and diagnosis of CeD are influenced by several factors, including demographics and genetics, and it often coexists with other autoimmune diseases. Thus, the objective of this paper was to review the literature on ophthalmic manifestations of CeD and to create preliminary considerations for these patients wishing to undergo elective corneal refractive surgery (CRS).

A literature review was conducted through July 2024, and relevant search terms were used to identify contributing papers. The review enabled the development of detailed considerations for the ocular manifestations of CeD and recommended management strategies for healthcare teams before and following CRS. The 24 papers included in this study illustrate that nutritional deficiencies and autoimmune concerns linked to CeD have distinctive ocular presentations. Based on these findings, patients with CeD may exhibit unconventional ocular manifestations beyond their typical gastrointestinal symptoms, such as decreased endothelial cell density (ECD), vitamin A deficiency leading to dryness, altered corneal nerve density, cataracts, uveitis, changes in choroidal thickness, papilledema, and neurological issues such as nystagmus. Patients with CeD may also experience synergistic impacts from concomitant autoimmune conditions such as Type 1 Diabetes Mellitus (T1DM) in addition to the coexistence of thyroid ophthalmopathy.

Recognizing that CeD is an autoimmune disorder that can be exacerbated by other conditions, it is essential to conduct a thorough evaluation for elective CRS. Due to the variability in ocular manifestations among CeD patients, individualized evaluations are crucial for determining surgical candidacy and optimizing outcomes, especially for patients who may not be well controlled. Evaluations are likely to encompass a subjective assessment through a questionnaire exploring the patient’s past medical history related to CeD. These questions can range from inquiring about general past medical history related to CeD regarding dietary gluten intake and weight loss to joint pain and cognitive impairments such as brain fog. Clinicians should also perform a comprehensive objective assessment utilizing slit-lamp biomicroscopy, Schirmer test, tear break-up time (TBUT), optical coherence tomography (OCT), Scheimpflug imaging, or fundoscopy.

Although there is currently no specific information regarding CRS recommendations for patients with CeD, we believe the considerations outlined in this paper should serve as preliminary guidelines for clinicians. While our findings have formed considerations for future healthcare teams, further research is needed to fully understand the impact of CeD on CRS outcomes and refine these recommendations.

## Introduction and background

Celiac disease (CeD), also known as celiac sprue, is a prevalent autoimmune disorder characterized by an abnormal immune response to gluten, leading to chronic enteropathy. Although it has been proposed that CeD was described by Aretaeus of Cappadocia, a second-century Greek physician, the first modern description of CeD was written in 1887 by the English physician Samuel Gee [1]. Current studies estimate the prevalence of biopsy-confirmed CeD to be approximately one in 133 individuals in the United States (U.S.) and around 0.7% globally [2,3].

However, the prevalence of CeD varies based on sex, age, familial history, immune status, and geographic location [3]. In the U.S., the mean age of diagnosis is 38 years, with a higher incidence observed in women [4]. CeD is typically diagnosed through serological tests, with IgA transglutaminase antibodies being the preferred initial screening method, followed by a confirmatory esophagogastroduodenoscopy (EGD) and duodenal biopsy. However, these tests may yield false-negative results in patients adhering to a gluten-free diet. Therefore, for undiagnosed patients, determining the individual's haplotype may be necessary due to the strong association of CeD with human leukocyte antigen-DQ2 (HLA-DQ2) and HLA-DQ8 [5].

As with many immunologic disorders, CeD frequently coexists with other systemic or autoimmune conditions [6]. These conditions may result from an increased predisposition for inflammatory processes and triggers within this patient population [7]. CeD is known for its various extraintestinal manifestations, among which ophthalmic presentations are particularly notable. These can encompass dry eye, uveitis, neuro-ophthalmic symptoms, and changes in several ocular measurements [8,9]. 

Recognizing the global rise in myopia [10], there is a growing likelihood that individuals seeking corneal refractive surgery (CRS) may also have CeD. Therefore, it is crucial to guide ophthalmologists on pertinent questions to ask during patient history taking and to highlight specific pathologies to focus on during ophthalmologic examinations. This paper aims to explore ophthalmic findings associated with CeD and provide a structured approach to assist clinicians in overseeing CeD patients considering CRS.

## Review

Methods

A literature search investigating the ophthalmologic manifestations of CeD was conducted using the databases PubMed, Scopus, and Ovid through July 01, 2024 with the following search term: “(celiac OR coeliac) AND (ocular manifestations OR ophthalmic manifestations OR anterior chamber OR cornea OR corneal OR LASIK OR photorefractive keratectomy OR laser-assisted in situ keratomileusis OR PRK OR small incision lenticule extraction OR SMILE) NOT (Review[Publication Type]) NOT (Systematic Review[Publication Type]) NOT (Meta-Analysis[Publication Type]).” The initial search yielded 110 results, of which 85 were excluded as irrelevant (39), non-English (10), books (five), and duplicates (31). Ultimately, 24 articles were included in the final analysis. Data extraction from the studies was performed independently by two reviewers who evaluated the range of ophthalmic manifestations encountered by patients with CeD and formulated considerations for CeD patients seeking CRS.

Results 

The 24 papers found through the literature search focused on the diverse ocular changes observed in patients with CeD. We categorized our findings on ocular manifestations resulting from CeD into distinct segments of the eye, encompassing the ocular surface, anterior segment, posterior segment, and neurological manifestations. Notable ophthalmic manifestations analyzed included dry eye, cataracts, uveitis, retinopathy, changes in choroidal thickness, papilledema, and nystagmus. 

*Ocular Surface* 

Ocular surface irregularities are often identified through standard corneal measurements such as central corneal thickness (CCT), corneal density, apex (CA), thinnest point (TP), and volume (CV). Studies by De Bernardo et al., Hazar et al., and Ozates et al. reported no significant differences in these measurements between CeD patients and normal controls [11-13]. However, another study found that endothelial cell density (ECD) was significantly lower in CeD patients (1765.78 ± 358.39 cells/mm² vs. 1906.31 ± 437.27 cells/mm²), indicating a potential pathological association [14]. Despite the increase in corneal Langerhans cells typically seen in autoimmune diseases like Type 1 Diabetes Mellitus (T1DM) and systemic lupus erythematosus (SLE), no such elevation was found in our review of CeD patients [15]. 

Studies have shown that dry eye is the most common ocular issue among individuals with CeD, often due to vitamin A and general nutritional deficiencies [9], thus making this ocular finding less pronounced in developed countries [16]. CeD patients compared to controls indicated decreased tear production and stability through the Schirmer test (11.33 ± 2.83 mm vs. 14.29 ± 2.91 mm) and tear break-up time (TBUT) (8.62 ± 2.74 s vs. 11.51 ± 2.31 s) [12,17]. 

Through impression cytology, one study found significant squamous cell metaplasia and goblet cell loss in CeD patients compared to controls, which is consistent with the finding of dry eye [17]. Xerophthalmia, a rare manifestation of vitamin A deficiency in CeD [18], can cause symptoms such as dryness of the conjunctiva and cornea, Bitot’s spots [19], and keratomalacia. These symptoms may lead to the loss of visual acuity, which can improve with vitamin A supplementation [20]. 

Additionally, there have been conflicting results regarding corneal nerve fiber integrity in CeD patients. Small nerve fibers, such as those in the cornea, can be affected due to reduced gut microbiota diversity, which may cause neuronal inflammation and damage [21,22]. It is theorized that a lack of gut microbiota diversity can lead to greater oxidative stress and neurodegenerative endotoxin formation as antibodies against gluten can cross-react with neuronal tissues, causing damage to glial cells [21,23,24]. However, data analyzed by Gad et al. showed no difference in corneal nerve fiber density or length between CeD children and healthy controls [25].

*Anterior Segment* 

Studies have demonstrated conflicting findings when assessing the pathological changes in the anterior segment in CeD patients with Scheimpflug imaging. One study found decreases in the anterior chamber angle (ACA) and anterior chamber depth (ACD) to values of 37.82 ± 5.91° and 2.76 ± 0.38 mm, respectively [14,26], whereas another study found these parameters to be increased to 42.21 ± 6.40° and 3.46 ± 0.31 mm, respectively [12]. Conversely, De Bernardo et al. found no differences in anterior chamber measurements in CeD patients compared to healthy controls [11].

Additionally, there have been varying reports of lenticular changes in CeD patients. Martins et al. found that cataract formation was prevalent in 12% of CeD patients, hypothesizing that chronic diarrhea due to CeD may induce osmotic changes in the lens, increasing its permeability and leading to its opacification [9]. They also theorized that cataract formation could be due to vitamin D and calcium deficiency, although none of their patients exhibited these deficits. Furthermore, prior findings have reported that although the mean lens density between CeD children and control patients is not different, the maximum lens density was significantly increased in CeD patients compared to controls (17.93 ± 7.16 vs. 15.24 ± 4.87 pixel intensity units) [13]. Ozates et al. suggested this could explain the early development of cataracts in CeD patients [13]. 

Uveitis is another ophthalmic manifestation that has been observed in CeD patients. Mollazadegan et al. reported that approximately 1% of their CeD patients developed uveitis, with a hazard ratio (HR) of 1.32 (95% CI 1.10 to 1.58) [27]. They also found that their risk estimate did not significantly change when adjusted for T1DM, rheumatoid arthritis, or autoimmune thyroid disease. However, other studies examining ophthalmic manifestations of CeD observed no signs of uveitis in their respective cohorts [17,28]. 

Posterior Segment

Several of the ophthalmic manifestations of CeD in the posterior segment have been associated with vitamin A deficiency. Sharma et al. described a malnourished pediatric patient who developed nyctalopia secondary to CeD [19]. Dotan et al. and Rani et al. reported that pediatric CeD patients with vitamin A deficiency developed papilledema and increased intracranial pressure (IIP), leading to the diagnosis of idiopathic intracranial hypertension (IIH), also known as pseudotumor cerebri. They also demonstrated that this condition resolved with the adoption of a gluten-free diet and Vitamin A/acetazolamide supplementation [29,30]. Vitamin A deficiency retinopathy has also been reported, in which CeD patients may present with retinal hypopigmentation, hemorrhage, and choroidal changes [31]. 

Other ophthalmic manifestations of CeD in the posterior segment may potentially be attributed to auto-antibody-mediated inflammation. Doğan et al. reported that CeD patients with seropositivity with anti-endomysial antibodies (anti-EmA) and anti-tissue transglutaminase type two antibodies (anti-TG2) had a thinner subfoveal choroidal thickness than non-seropositive CeD patients, although their findings were statistically insignificant and the authors could not prove the presence of autoantibodies in the choroidal microcirculation [32]. However, another study demonstrated that CeD patients had thicker choroids than healthy controls (372.38 ± 92.82 µm vs. 315.51 ± 86.63 µm), regardless of the corresponding axial length [33]. CeD patients also showed a significantly thinner central macular thickness (CMT), 217.83 ± 14.28 μm in CeD patients and 222.40 ± 12.63 μm in healthy controls [14]. A study by Hazar et al. also found that higher anti-tTG IgA levels were correlated with a thicker nasal retinal nerve fiber layer (RNFL) and a thinner superior RNFL [12], and Dönmez Gün et al. added that prolonged gluten-free diets were associated with a thinner mean and inferior RNFL [14].

CeD patients who are concurrently diagnosed with T1DM are also at an increased risk for the development of diabetic retinopathy. One study showed that CeD patients with T1DM had a significantly higher prevalence (58%) than a randomly selected and matched control group of T1DM-only patients (25%) [34]. Furthermore, Vogt-Koyanagi-Harada (VKH) syndrome, an autoimmune condition targeting melanocytes, has been scarcely observed in one patient with CeD and T1DM. It can lead to decreased visual outcomes associated with exudative retinal detachment and retinal pigment atrophy [35]. This further substantiates the relationship between CeD and additional autoimmune conditions that often present with ocular defects. 

Neurological Manifestations

Neurological manifestations can occur in CeD, presenting as gluten ataxia [36], or in rare cases, opsoclonus [37]. These changes are proposed to be from autoantibodies and independent of associated vitamin deficiencies. Our findings underscore gluten ataxia as a prominent neurological symptom observed, as reported in two studies [36,38]. In patients with gluten ataxia, ocular manifestations typically present as nystagmus and difficulties in pursuit of eye movements [36]. Opsoclonus-myoclonus syndrome (OMS) will present as irregular, high-frequency saccades that are multidirectional, accompanied by involuntary jerks of the limbs and trunk [37,39,40]. These findings were reported in two cases, including a pediatric patient whose OMS improved following a gluten-free diet over a period of four weeks [37,38]. 

Discussion 

Our review provides clinicians with preliminary insights into preoperative screening and potential impacts on the CRS outcomes of patients with ocular manifestations of CeD, improving outcomes for those considering CRS. The initial approach involves a subjective examination of CeD’s numerous systemic effects once the patient discloses their CeD diagnosis (Tables 1, 2). After completing the subjective assessment, patients should undergo an objective ophthalmic examination tailored to the potential ocular presentations observed in individuals with CeD. 

**Table 1:** Subjective systemic assessment of CeD patients prior to CRS Abbreviations: CeD = celiac disease; CRS = corneal refractive surgery

Past Medical History Assessment (Systems Based)	Questionnaire
General	Are you on a strict gluten-free diet (wheat, rye, and barley)?
	History of any autoimmune conditions or chronic diseases?
	Unexplained weight loss or poor weight gain?
	Fatigue or failure to thrive?
Central Nervous System (CNS)	Gait imbalance?
	Tingling, numbness/neuropathy?
	Depression or anxiety?
	Brain fog?
Gastrointestinal	Bloating, gas, and/or abdominal pain?
	Diarrhea or constipation?
	Nausea or vomiting?
Musculoskeletal	Joint pain?
	Muscle pain or stiffness?
Dermatological	Itchy skin or rash?
	Discolored teeth or loss of enamel?

**Table 2:** Subjective ophthalmic assessment for CeD patients prior to CRS Abbreviations: CeD = celiac disease; CRS = corneal refractive surgery

Ophthalmic History Assessment	Questionnaire
Vitamin A Deficiency	Night blindness?
	Dry eye?
	Skin dryness or thickening?
Uveitis	Redness?
	Light sensitivity?
	Prior uveitis? Recurrence?
Cataracts	Vision changes including clouding or blurring?
	Color changes or dimming?
	Difficulty seeing at night?
Nystagmus	Dizziness or balance issues?
	Reduced visual sharpness?
Thyroid-associated Orbitopathy	Pain in or behind the eye?
	Bulging eyes?
	Excessively dry or watery eyes?
	Difficulty closing or moving eyes?

Due to the increased predisposition of dry eye in CeD patients [9,17], clinicians should inquire about related symptoms during the ophthalmic workup for CRS. A detailed biomicroscopy exam can be used to confirm the presence of dry eye disease and superficial punctate keratitis. Additionally, other diagnostic tests such as ocular surface staining with fluorescein, Schirmer test, and TBUT are recommended to confirm dry eye disease (Table 3). CeD patients who present with symptoms such as ocular discomfort, irritation, redness, burning, and decreased visual acuity due to dry eye disease [12] can be managed with artificial tear supplementation and/or punctal plug implementation prior to undergoing CRS. 

**Table 3:** Comprehensive objective ophthalmic assessment for CeD patients prior to CRS Abbreviations: = CeD = celiac disease; CRS = corneal refractive surgery; ACE = angiotensinogen converting enzyme; HLA = human leukocyte antigen; HPLC = high performance liquid chromatography; OCT = optical coherence tomography; RNFL = retinal nerve fiber layer

Workup	Potential Findings	Testing
General	Celiac Disease	Anti-endomysial antibody (EmA-IgA)
		Anti-tissue transglutaminase antibodies (tTG) (tTG-IgA) (tTG-IgG)
		HLA testing: HLA-DQ2 and HLA-DQ8
Nutrition Panel: Vitamin A	Vitamin A deficiency	Serum retinol HPLC
		Serum retinol spectrometry
Autoimmune Comorbidity	Type 1 Diabetes Mellitus	Insulin autoantibodies (IAA)
	Intermediate Uveitis	Serum ACE and lysozyme
	Anterior Uveitis	HLA-B27, Serum ACE and lysozyme
External/ Adnexa	Nystagmus	Visual inspection/ Optokinetic drum
	Thyroid-associated orbitopathy	Hertel exophthalmometer
Ocular Surface	Decreased Endothelial Cell Density	Specular microscopy
	Dry eye	Fluorescein surface staining
		Schirmer Test
		Impression cytology
		Slit-lamp biomicroscopy
	Bitot's spots	External examination
	Keratomalacia	
	Corneal Neuropathy	Confocal Microscopy
Anterior Segment	Decreased Anterior Chamber Depth	Scheimpflug imaging and biometry
	Signs of early onset cataracts	Slit-lamp biomicroscopy
Posterior Segment	Changes in choroidal thickness	OCT
	Central macular thickness	
	Decreased thickness of RNFL	Fundoscopy
	Papilledema	

Ocular measurements in CeD patients can be distinct from those in the general population due to various yet unknown pathogenic mechanisms. Previous research on ocular changes in CeD patients has concentrated on specific parameters including ECD, ACD, choroidal thickness, and CMT. ECD, in particular, has been found to be a vital corneal metric with adverse effects such as corneal edema following a clinically significant reduction in endothelial cells. Although prior studies indicate a statistically significant decrease in endothelial cell count in normal patients who underwent laser-assisted in situ keratomileusis (LASIK) and small incision lenticule extraction (SMILE) procedures six months post-surgery [41], it is important to note the observed reduction in ECD in patients with CeD [14]. Realizing that patients with CeD may already have a lower ECD prior to refractive surgery, it is crucial to perform specular microscopy in these patients before proceeding with CRS [14]. If a clinician has a high concern or clinical suspicion of reduction of the ECD, then the acquisition of such a diagnostic test is recommended despite its unavailability in certain eye care facilities. 

Clinicians should evaluate ACD in CeD patients, as some studies indicate a potential for shallower depths in this population [14]. Furthermore, shallow ACDs in myopes, in addition to a significantly increased axial length, might suggest early-onset cataracts [41]. Knowing that the current literature demonstrates varying results concerning this parameter in CeD patients, we believe that it is still important for clinicians to use the Scheimpflug imaging and biometry to evaluate ACD. While patients considering CRS typically belong to a younger age group and are not expected to present with cataracts, it is important to note that studies report the increased likelihood of early cataracts in CeD patients, potentially due to osmotic changes and/or nutritional deficiencies [27]. Therefore, a thorough evaluation for cataracts with slit-lamp biomicroscopy should not be overlooked in the preoperative assessment of these patients (Table 3). 

A comprehensive evaluation using optical coherence tomography (OCT) is also crucial for patients with CeD to gain insights into choroidal thickness and structure as well as CMT abnormalities (Table 3). Acknowledging the variation in choroidal thickness in CeD patients, the primary concern is that those patients with a thinner subfoveal choroid, specifically 30 μm or thinner, have been reported to exhibit lower best-corrected visual acuity (BCVA) [42]. Furthermore, changes in choroidal thickness can lead to potential complications such as choroidal neovascularization and central serous chorioretinopathy, increasing the risks of perioperative bleeding and poor postoperative visual potential, respectively [43]. Additionally, despite successful surgical outcomes and an unremarkable anterior segment, patients with posterior segment pathological findings, such as reduced CMTs or choroidal thickness, may still experience diminished visual acuity [44].

The coexistence of T1DM and CeD raises significant concerns regarding diabetic retinopathy, an important complication of diabetes that can severely impact vision. Previous research has indicated that, due to shared pathogenic mechanisms, individuals with T1DM are more likely to develop CeD compared to the overall population (5% vs. 1%) [45]. CRS in CeD patients with this presentation may lead to post-surgical site infection [46] due to uncontrolled hyperglycemia [47]. At the same time, studies show that patients with diabetic retinopathy may present with decreased corneal sensation [48]. Therefore, these patients can have a higher risk of diabetic keratopathy, which may impair wound healing during the process of CRS [49]. 

When T1DM is suspected in patients with CeD, stringent protocols should be adopted to assess the retina's integrity and overall ocular health due to the impact on the RNFLs [12,14]. An assessment of the retina can be conducted through a comprehensive evaluation of the posterior segment, which can be evaluated for retinopathy as well as thinning of the RNFLs and macula (Table 3). 

Additionally, it is noteworthy that procedures such as SMILE, LASIK, and even photorefractive keratectomy (PRK) can have a direct or indirect impact on the corneal nerve plexus and nerve fiber endings, increasing the risk of postoperative dry eye [50,51]. Therefore, the literature on CeD suggests that assessing the subtle changes in corneal nerve integrity through confocal microscopy may be warranted for CeD patients undergoing CRS [52]. However, it is important to note that confocal microscopy may not be readily available in clinical practice. Nevertheless, utilizing this approach, if feasible, will ensure a more comprehensive screening and reduce the risk of postoperative dry eye. 

Recognizing the autoimmune nature of CeD, it is evident that CeD patients have a higher risk of developing uveitis [53-55]. Intriguingly, there is a documented instance where uveitis has responded favorably to a gluten-free diet in CeD patients, suggesting a direct link between diet management in CeD and the mitigation of uveitis symptoms [53]. Given the impact of uveitis on postoperative visual potential and prognosis, it is recommended to thoroughly screen and ensure no documented presence of uveitis for at least 12 months before elective CRS [56,57]. Comprehensive pre-surgical evaluations, improved gluten control, and a complete uveitis workup are essential if patients have had a prior history of uveitis (Table 3). 

Thyroid-associated orbitopathy (TAO) has also been associated with CeD [58]. If suspected, a series of diagnostic questions can be posed (Table 2). Inflammation from TAO can lead to proptosis, causing corneal exposure, increased astigmatism, higher corneal epithelium permeability, and reduced nerve density [59,60]. Moreover, individuals diagnosed with thyroid eye disease and TAO have been shown to be biomechanically and morphologically similar to keratoconus patients [59,61]. Given that keratoconus is an explicit exclusion for CRS, the eligibility of CeD patients with TAO for CRS should be investigated. If patients have reported CeD, it is imperative to assess a patient's thyroid status through an orbital ultrasound. Should thyroid dysfunction be identified, a thyroid function test and consultation with their endocrinologist are essential. CRS should only be performed if the patient’s thyroid condition is stable. 

Vitamin A deficiency, often discernible through specific clinical presentations, can be substantiated by an external ocular examination to assess for dryness (Table 2) and biochemical markers (Table 3). Serum retinol levels below 20 µg/dL indicate a deficiency [62] and should prompt physician referral. Management of vitamin A deficiency generally involves supplementation, leading to the timely resolution of symptoms such as corneal lesions and Bitot’s spots, with retinal issues such as night blindness taking slightly longer [63]. Specific supplementation dosages of vitamin A include 10,000 IU daily for three months [63]. It is crucial to communicate with a primary care doctor for management, especially if their vitamin A levels are abnormal.

However, in patients not receiving supplementation, further ophthalmic evaluations are crucial to prevent the progression of symptoms, which can result in the loss of visual acuity [64]. Vitamin A deficiency has also been found to delay wound healing, an important consideration after surgery, as vitamin A plays a crucial role in collagen production [65]. For CeD patients who were not receiving vitamin A supplementation, a cost-effective serum retinol spectrometry should be initiated. Should clinical indicators of Vitamin A deficiency be present, high-performance liquid chromatography (HPLC) for serum retinol testing should be employed as it offers greater reliability [62].

Additionally, patients with CeD may develop papilledema, a strong contraindication for CRS. Therefore, a thorough fundoscopic examination of the optic nerve for careful scrutiny of its anatomical landmarks is imperative to identify signs of an edematous optic disc, such as elevated and blurred disc margins, prior to the initiation of CRS (Table 3). In severe cases, the optic disc may be completely obliterated by swelling on evaluation [66].

Questions about seemingly unrelated symptoms, such as joint pain, cognitive dysfunction, and gait imbalance are important because CeD can cause several extraintestinal manifestations, despite unclear mechanisms (Table 1). Although rare, CeD patients may have gluten ataxia, which can exhibit neurological manifestations such as gait ataxia and nystagmus, as reported in 11% of newly diagnosed CeD patients [67,68]. CRS can be challenging or a relative concern in patients with nystagmus due to the lack of complete motor control, which may impact eye tracking during the laser excimer ablation. This can potentially cause problems during the ablation phase of refractive surgery.

## Conclusions

In this literature review, we examined the ocular manifestations of CeD and evaluated the considerations and additional assessments necessary for CeD patients contemplating CRS. Our findings suggest a comprehensive ophthalmic workup is essential to identify any preexisting ocular conditions in CeD patients that may contraindicate CRS. Given the variability in the severity of ocular manifestations among CeD patients, individualized evaluations are crucial to ensuring appropriate surgical candidacy and optimizing outcomes. We must also employ targeted scrutiny to identify unique clinical findings or ask specific questions that can better detect the subset of CeD patients who may not be well-controlled and are interested in CRS.

While much remains to be elucidated regarding the effects of CeD, our current findings have initiated the formulation of guidelines for future clinicians when managing CeD patients seeking CRS. The recommendations provided should be viewed as a preliminary framework, underscoring the necessity for further research directly examining the impact of CeD on CRS outcomes.
